# Rectal mucosal prolapse syndrome as an unusual gastrointestinal manifestation of Sjögren's syndrome: a case report

**DOI:** 10.1186/1752-1947-3-85

**Published:** 2009-10-30

**Authors:** Hideki Koga, Kayoko Shimizu, Ken-ichi Tarumi, Yoshito Sadahira, Takayuki Matsumoto, Mitsuo Iida, Ken Haruma

**Affiliations:** 1 Department of Medicine and Clinical Science, Graduate School of Medical Sciences, Kyushu University, Maidashi, Higashi-ku, Fukuoka 812-8582, Japan; 2 Division of Gastroenterology, Department of Medicine, Kawasaki Medical School, Japan; 3 Department of Pathology, Kawasaki Medical School, Matsushima, Kurashiki 701-0192, Japan

## Abstract

**Introduction:**

Rectal mucosal prolapse syndrome, histologically characterized by fibromuscular obliteration in the lamina propria, hyperplastic glands and thickened muscularis mucosa, causes rectal bleeding. Sjögren's syndrome is an autoimmune exocrinopathy that chiefly destroys the salivary and lacrimal glands by lympho-plasmacytic infiltration. Although various gastrointestinal manifestations have been reported in patients with Sjögren's syndrome, there have not been to our knowledge any case reports to date of rectal mucosal prolapse syndrome in association with Sjögren's syndrome.

**Case presentation:**

A 68-year-old Japanese woman with Sjögren's syndrome and long-term constipation consulted our hospital because of rectal bleeding. Because of dysphagia and xerostomia, she had consistently refused recommendations to take oral medicines including cathartics. Therefore, she frequently strained excessively during defecation. Colonoscopy and radiological examinations disclosed eroded flat protrusions of the rectum. Microscopic examination demonstrated inflamed mucosa with elongated tortuous glands and fibromuscular obliteration. Based on these findings, a diagnosis of rectal mucosal prolapse syndrome was made. Prohibition of straining during defecation and sulfasalazine suppository use were effective.

**Conclusion:**

This case highlights the importance of defecation control in patients with Sjögren's syndrome. In the case presented, rectal mucosal prolapse syndrome following long-term excessive straining during defecation caused rectal bleeding. Clinicians should consider rectal mucosal prolapse syndrome as a gastrointestinal manifestation of Sjögren's syndrome.

## Introduction

Rectal mucosal prolapse syndrome (RMPS), which is histologically characterized by fibromuscular obliteration in the lamina propria, hyperplastic glands and thickened muscularis mucosa [1], shares its clinicopathological basis with solitary rectal ulcer syndrome [2], colitis cystica profunda [3] and cap polyposis [4,5]. Because the gross appearance of RMPS varies, its diagnosis is often delayed.

Sjögren's syndrome (SjS) is an autoimmune exocrinopathy that chiefly destroys the salivary and lacrimal glands by lympho-plasmacytic infiltration [6]. Although various gastrointestinal manifestations, especially esophagogastric disorders, have been reported in patients with SjS [7], there have never been, to our knowledge, any case reports of RMPS in association with SjS.

We examined and treated a patient with SjS who, in spite of severe constipation, had never taken cathartics for fear of worsening of an SjS-induced swallowing disturbance and who finally developed RMPS related to long-term excessive straining during defecation. Here, we describe in detail what we believe is the first reported case of PMRS in association with SjS.

## Case presentation

A 68-year-old Japanese woman consulted our hospital in October 2005 because of repeated rectal bleeding. A diagnosis of SjS had been made 15 years before because of severe xerostomia and dry eye. Anti-Ro/SSA and -La/SSB antibodies were both positive. The patient had had persistent constipation for several years. At her local clinic, she was repeatedly advised to take oral medicines such as cathartics or laxatives. However, she consistently refused these recommendation because she had difficulty in swallowing medicine due to xerostomia. Therefore, she had developed a habit of straining during defecation.

**Figure 1:**
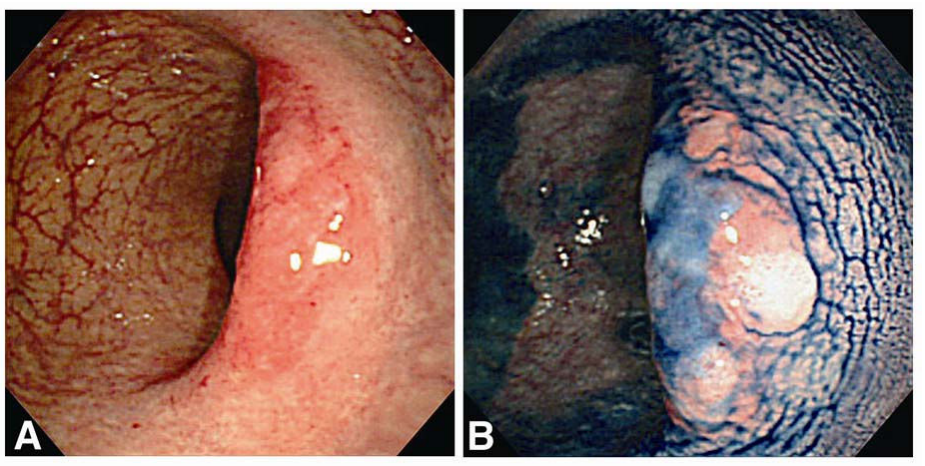
**Initial colonoscopy findings**. An initial colonoscopy revealed reddened, flat-elevated patchy lesions in the rectum (A). After spraying dye, a shallow and large ulceration was clearly noted (B).

Colonoscopy disclosed reddened flat-elevated lesions in the rectum. A patchy erythematous lesion was observed on the right wall of the proximal rectum, and a similar lesion was seen on the left wall of the distal rectum just above the anal verge (Figure 1A). On the top of both lesions, a large shallow ulceration became clearer with a chromocolonoscopy after spraying of indigo-carmine dye (Figure 1B). A subsequent barium enema examination demonstrated large but subtle lesions in the rectum. One lesion showed increased wall thickness with a faint barium fleck, and another showed a nodular flat protrusion with slight deformity of the wall.

Microscopic examination of biopsy specimens revealed inflamed mucosa with elongated tortuous glands attenuated toward the mucosal surface. Fibromuscular obliteration typical of mucosal prolapse syndrome was also observed. This inflamed mucosa, however, was not covered with a cap of inflammatory granulation tissue. The intervening mucosa was histologically normal.

**Figure 2:**
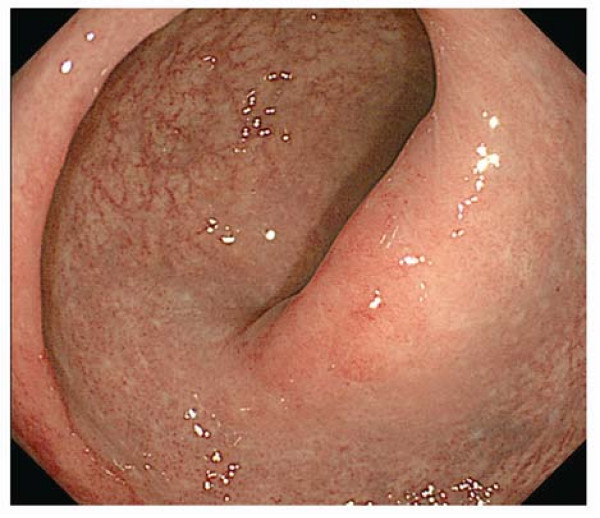
**The second colonoscopy findings**. Three months after medical treatment and alteration in defecation habit, the rectal ulceration had disappeared and mucosal inflammation had improved.

Based on these clinicopathological findings, a diagnosis of RMPS was made. We advised the patient strongly not to strain during defecation, and administered a 500 mg sulphasalazine suppository daily. Three months later, a follow-up colonoscopy disclosed disappearance of the ulceration and marked improvement of the rectal inflammation (Figure 2).

## Discussion

Within the gastrointestinal tract, the esophagus and the stomach are the sites most commonly involved in SjS [8]. Dysphagia, which is one of the most common symptoms of SjS, is thought to be the result of esophageal dysmotility or webs [8]. Chronic atrophic gastritis is also common in patients with SjS [9]. Regarding the lower gastrointestinal tract, there have been some reports of SjS associated with small bowel diseases. Celiac disease, or gluten-sensitive enteropathy, is more frequent in patients with SjS than in the average healthy population [10]. Recently, Krogh et al. [11] reported a significantly higher prevalence of bowel symptoms including constipation in patients with SjS. However, there have been only a few reports of SjS involving the colorectum. Two cases of colonic cancer associated with SjS, one sigmoid colon cancer [12] and one cecal cancer [13], have been reported, but the pathognomonic relationship between SjS and colonic cancer remains unclear. To date, to the best of our knowledge, there have never been any reports of SjS associated with RMPS.

In SjS, gastrointestinal dysmotility often develops. Esophageal manometric studies have disclosed absent or decreased contractility in the upper third of the esophagus, spontaneous biphasic and triphasic tertiary contractions in the lower two thirds of the esophagus, aperistalsis, frequent nonperistaltic contraction and low amplitude contraction [8]. Gastric emptying scintigraphy using ^99m^Tc-human serum albumin has revealed gastric emptying to be abnormally slow in 70% of the examined patients with SjS [14]. Adachi et al. [15] reported a case of chronic intestinal pseudo-obstruction with SjS. These reports suggest there may be an autonomic nervous system dysfunction involving the gastrointestinal tract in SjS [14].

Unfortunately, the gastrointestinal motility function in our patient was unclear because she had refused to undergo any gastrointestinal motility examination. Considering the long-term severe constipation experienced by our patient, it may be reasonable to suppose that our patient also previously had colorectal dysmotility. Interestingly, a barium enema examination demonstrated diverticula in both her left and right colon. Diverticular disease of the colon seems to be a multifactorial condition, and a recent study disclosed the pathognomonic role of smooth-muscle dysfunction resulting from autonomic nerve attrition in diverticulosis [16]. Therefore, in our patient, abnormality of the gastrointestinal autonomic nervous system associated with SjS may have caused dysmotility of the intestine, resulting in severe constipation and colonic diverticula. Despite uncontrollable constipation probably derived from dysmotility of the colorectum, our patient had never taken any cathartic because of xerostomia and dysphagia.

## Conclusion

Our case highlights the importance of defecation control in patients with Sjögren's syndrome. It may be necessary to recognize RMPS as a rare but important complication of SjS.

## Abbreviations

RMPS: rectal mucosal prolapse syndrome; SjS: Sjögren's syndrome.

## Competing interests

The authors declare that they have no competing interests.

## Authors' contributions

HK performed the colonoscopy examination and was a major contributor in writing the manuscript. KS collected the patient data regarding Sjögren's syndrome. KT analyzed the colonoscopy findings of the patient. YS performed the histological examinations of the biopsy samples taken from the rectum during the colonoscopy examinations. TM, MI and KH made appropriate suggestions during the preparation of the manuscript. All authors read and approved the final manuscript.

## Consent

Written informed consent was obtained from the patient for publication of this case report and accompanying images. A copy of the written consent is available for review by the Editor-in-Chief of this journal.
